# The complete mitochondrial genome of an edible mushroom, *Sparassis crispa*

**DOI:** 10.1080/23802359.2020.1715855

**Published:** 2020-01-27

**Authors:** Khawaja Muhammad Imran Bashir, Kyoung Min Rheu, Moo-Sang Kim, Man-Gi Cho

**Affiliations:** a German Engineering R&D Center for Life Science Technologies in Medicine and Environment, Busan, Republic of Korea;; b Department of Biotechnology, Division of Bio-Chemical Engineering, Dongseo University, Busan, Republic of Korea;; c Digital Omics, Daejeon, Republic of Korea

**Keywords:** Mitogenome, next-generation sequencing, *Sparassis crispa*, whole genome sequencing (WGS)

## Abstract

*Sparassis crispa,* also known as cauliflower mushroom, is a widely used medicinal mushroom in traditional Chinese medicine due to the presence of bioactive substances with pharmacological activity. Here, we report a complete mitochondrial genome sequence of *S. crispa* consisting of 139,253 bp containing 47 genes including 15 protein-coding genes, 27 transfer RNA, and 5 ribosomal RNA genes obtained from 40.406 Mb genome containing 18,917 predicted contigs using raw data of next-generation sequencing having 85.4% Q30. The overall base composition of *S. crispa* was 26.47% G-C and 73.53% A-T. The phylogenetic tree based on *atp6* sequence data showed its close relationship with *Sparassis radicata*. The complete mitochondrial genome sequence of *S. crispa* provides an essential and important DNA molecular data for further phylogenetic and evolutionary analysis of *S. crispa*.

The brown-rot producing cauliflower mushroom, *Sparassis crispa,* is widely distributed in Australia, North America, Europe, and East Asia (Martin and Gillbertson 1976). *Sparassis crispa* mainly grows on coniferous trees such as *Larix kaempferi, Pinus densiflora, Pinus koraiensis* (Lee et al. 2015). It is known for its medicinal significance due to the availability of various pharmacological substances and their use in health supplements (Kimura 2013; Bashir and Choi 2017). Previous studies have reported the phylogenetic analyses of mushrooms using nucleotide sequence data from ribosomal DNAs, partial RNA polymerase subunit II gene, and mitochondrial ribosomal DNA (Dai et al. 2006; Ryoo et al. 2013; Kiyama et al. 2018; Xiao et al. 2018). Among the eight reported clades in *Sparassis spp.* [*Sparassis brevipes, Sparassis crispa, Sparassis cystidiosa, Sparassis latifolia, Sparassis miniensis, Sparassis radicata, Sparassis spathulate, Sparassis subalpina*], the Asian collections of *S. crispa* are morphologically different from the European collections; thus it is important to identify this mushroom at the level of mitochondrial DNA.

Here, we report the complete mitochondrial genome of *S*. *crispa* using next-generation sequencing, which will help to better understand the phylogenetic status of *S. crispa*. *Sparassis crispa* was obtained from the Culture Collection of Wild Mushrooms, Incheon, Republic of Korea (37.46 latitude, 126.67 longitude). This specimen is stored at the Culture Collection of Wild Mushrooms, Incheon, Republic of Korea [Reference No. IUM04050]. The mycelium was grown in the laboratory as described previously by Lee et al. (2015) with slight modifications. Briefly, the fruiting bodies of *S. crispa* were sterilized, isolated, and cultured at 23 ± 2 °C on potato dextrose agar medium. The mitochondrial DNA was sequenced using Illumina Hiseq X sequencing (Illumina 2014). The genome assembly was done using CLC assembly 5.1.1 (Qiagen, Aarthus C, Denmark) and the annotation of full sequence was performed using BLAST, tRNAscan-SE2.0 (Lowe and Chan 2016), and MITOS webserver (Bernt et al. 2013; Han et al. 2018).

Length of the complete mitochondrial genome of *S. crispa* was 139,253 bp containing 47 genes including 27 transfer RNA genes, 15 protein-coding genes of seven NADH dehydrogenases (*nad1*, *and2*, *nad3*, *nad4*, *nad4L*, *nad5*, and *nad6*), three ATPases (*atp6*, *atp8*, and *atp9*), three cytochrome c oxidases (*cox1*, *cox2*, and *cox3*), an apocytochrome b (*cob*), and a ribosomal protein S3 (*rps3*), and 5 ribosomal RNA genes (three *rns* and two *rnl*), obtained from 40.406 Mb genome containing 18,917 predicted contigs using raw data of next-generation sequencing having 85.4% Q30 (ratio of bases having Phred quality score of *>*30). The complete and annotated mitochondrial genome of *S. crispa* has been deposited to GenBank [accession number: MN722635].

To predict the phylogenetic evaluation of *S. crispa*, a neighbor-joining phylogenetic tree was constructed with MEGA7 (Tamura et al. 2013), using *atp6* of representative species belonging to *Ascomycota* or *Basidiomycota*, especially, *S. radicata* (Figure 1). The phylogenetic analysis with other fungal genomes did not show much difference, suggesting that the previously reported *S. radicata* (Dai et al. 2006) and currently reported *S. crispa* are very close to each other.

**Figure 1.**
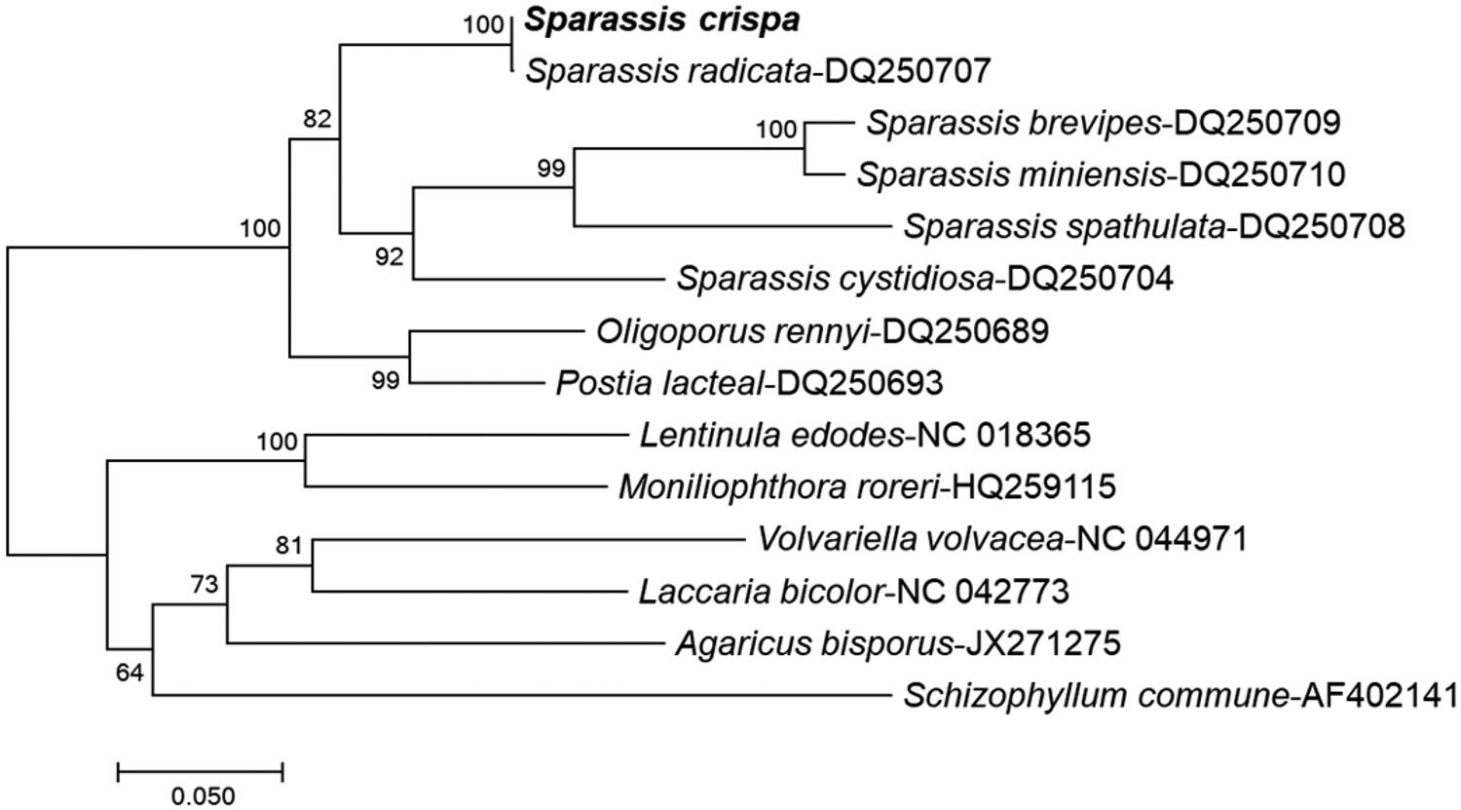
Phylogenetic tree of *S. crispa* with other closely related fungal species. The tree was constructed by neighbor-joining method based on the *atp6* nucleotide sequence using MEGA7 software. Bootstrap test data of 1000 replicates showing less than 70% site coverage were eliminated.
